# Isolation, Screening, and Characterization of Antibiotic-Degrading Bacteria for Penicillin V Potassium (PVK) from Soil on a Pig Farm

**DOI:** 10.3390/ijerph16122166

**Published:** 2019-06-19

**Authors:** Xuanjiang Yang, Miao Li, Panpan Guo, Hualong Li, Zelin Hu, Xianwang Liu, Qiang Zhang

**Affiliations:** 1 Institute of Intelligent Machinery, Hefei Institute of Material Sciences, Chinese Academy of Sciences, Hefei 230031, Anhui, China; xjyang@mail.ustc.edu.cn (X.Y.); panpan2019520@163.com (P.G.); hlli@iim.ac.cn (H.L.); zlhu@iim.ac.cn (Z.H.); lxw440@163.com (X.L.); 2 Department of Biosystems Engineering, University of Manitoba, Winnipeg, MB R3T 5V6, Canada; Qiang.Zhang@umanitoba.ca

**Keywords:** penicillin, biodegradation, soil, pig farm, bacterial isolate, *Bacillus*

## Abstract

(1) Background: Antibiotics are frequently used on farm animals, making animal husbandry a relatively large source of antibiotic pollution of the environment. The present study aims to isolate and acclimatize antibiotic-degrading bacterial strains for penicillin V potassium (PVK) from the contaminated soil of a pig farm. (2) Methods: Bacterial strains were isolated and acclimatized by continuous enrichment of cultures with PVK as the sole carbon source. The antibiotic susceptibility test, thiol mercury salt ultraviolet spectrophotometry (TMSUS), morphological observations, and 16S rDNA sequence analysis were used to identify and characterize the isolated strains. (3) Results: Four bacterial isolates (denoted as LM-1, LM-2, LM-3, LM-4) were obtained, and two of them (LM-1, LM-2) with the highest degradation rates were identified to belong to the same genera as *Bacillus*. These two isolates were found to be resistant to PVK antibiotic in an antibiotic sensitivity test. The TMSUS indicated that the strains LM-1 and LM-2 had good performance in PVK degradation (68% for LM-1, 66% for LM-2 in 48 h) when the initial PVK concentration was about 100 μg/mL. (4) Conclusions: Two bacterial strains isolated from the soil on a pig farm are effective in degrading PVK and can be potentially used for bioremediation of PVK antibiotic-contaminated soils.

## 1. Introduction

Intensive livestock farming has been identified to be associated with extensive usage of antibiotics contained in pharmaceuticals that are widely produced and applied to treat bacterial infections in humans and livestock [1,2,3]. The use of antibiotics in livestock production is forbidden in some regions in the world, (e.g., the European Union since 2006) because of the overuse, and misuse or lack of control in administering antibiotics, resulting in the accumulation of antibiotics in animal products and the environment [4,5]. Research has shown that the spread of multidrug resistant bacterial strains and their potential adverse effects on animals and environment are closely associated with the abuse of antibiotics [6,7]. Numerous studies have reported the detection of antibiotics in coastal water and wastewater at concentrations from μg/L to mg/L levels [8]. Furthermore, antibiotic resistant genes harbored by mobile genetic elements have proven to be transferred between bacteria and the environment [9,10]. The potential epidemiological risks to human health triggered by horizontal antibiotic resistant gene transfer will increase with antibiotic resistance proliferation.

A group of commonly used antibiotics that attack a wide range of bacteria are penicillins, such as penicillin V potassium (PVK), which is a slow-onset antibiotic that is useful for the treatment of a number of bacterial infections. Although it is less active than other drugs, like penicillin G, against Gram-negative bacteria, PVK is more acid-stable than natural penicillins, allowing it to be taken orally without being decomposed by gastric acid. Furthermore, PVK is designed to have a good hydrolysis-resistant capability to strengthen the stability in treating bacterial infections [11]. Substantial concentrations of PVK have been found in livestock manure, soil, and wastewater effluents, which may pose potential threats to human health and contribute to the emergence of penicillin-resistant bacterial strains. Therefore, it is imperative for researchers to find ways to degrade the residues of PVK in the environment.

Various methods have been explored to remove penicillin antibiotics from wastes, including chemical hydrolysis, membrane separation, activated carbon adsorption, and photodegradation. However, there are a number of drawbacks associated with these methods, including the formation of secondary toxic byproducts and high operational costs, when using the conventional physical and chemical techniques [12]. Biodegradation may provide alternative tools to degrade antibiotics in wastes. Zhao et al. [13] reported that a highly chelate coccus PC-2, which could use penicillin sodium as the sole carbon source, was screened and separated from the compost materials containing penicillin bacteria residues mixed with pig manure. Adel et al. [14] reported that significant degradation of cephalexin antibiotics was produced by the *Bacillus subtilis* strain. Al-Gheethi et al. [15] investigated the biodegradation by *Bacillus subtilis* of four *β*-lactam antibiotics (amoxicillin, ampicillin, cefalexin, cefuroxime) and found that *Bacillus subtilis* treatment was especially efficient for removing amoxicillin, ampicillin, and cefalexin at a concentration of 1 mg/L, with removal rates up to 25.03%, 22.59%, and 10.62%, respectively.

The goal of this study is to find bacteria, naturally existing in soil for degradation of antibiotics. The specific objectives are to: (1) Isolate and characterize bacteria form soil on a pig farm for degradation of PVK, and (2) evaluate the effectiveness of the isolated bacterial strains. PVK was selected in this study not only because it is a class of penicillin antibiotics that are extensively used in both livestock production and human infection treatment, but also due to its chemical structure—PVK may serve as the sole carbon and energy source for the bacterial isolates, which can be identified and characterized by using phylogenetic analysis of 16S rDNA sequences.

## 2. Materials and Methods

The enrichment and acclimatization method was used for isolating and screening PVK-degrading bacterial strains. The isolated bacterial strains were then quantitatively evaluated for their effectiveness in degrading PVK by using the thiol mercury salt ultraviolet spectrophotometry (TMSUS). The selected high-performance strains were then identified by 16S rRNA gene sequencing and phylogenetic analysis. The laboratory approach used in this study was similar to those reported in the literature, such as Liu [16] and Feng [17], which have been proven to be reliable in screening the antibiotic-degrading bacterial strains.

### 2.1. Collection of Soil on Pig Farm

Soil extracts were collected from a pig farm in Hefei, China. This farm was selected because there were PVK residuals in the soil from pig manure, which naturally led to the emergence of bacteria that feed on PVK. A total of 1000 g of soil was gathered from the east, south, west, north sides, and the center of the farm. Collected soil was placed in sealed sterile bags and kept at a temperature above −20 °C before the experiment.

### 2.2. Reagents and Culture Media

The target antibiotic, penicillin V potassium (PVK, purity ≥ 99.0%) used in this study, was purchased from Sigma-Aldrich (St. Louis, MO, USA). Boric acid (analytical purity ≥ 98.0%), Ni-acetonitrile (chromatographic purity), Ni-acetic anhydride (analytical purity ≥ 99.0%), hydrochloric acid (analytical purity ≥ 99.0%), sodium hydroxide (analytical purity ≥ 99.0%), imiparzole (analytical purity ≥ 99.0%) were purchased from a commercial source (Bangzhili Co., Ltd., Hefei, China). The Luria–Bertani (LB) medium used for enrichment of the bacterial strains contained NaCl (5 g/L), yeast extract (5 g/L), and tryptone (10 g/L) at pH 7.0. The mineral salt medium (MSM) used for acclimatization of bacteria strains contained 0.45 g/L KH_2_PO_4_, 0.1 g/L MgSO_4_·7H_2_O, 1.79 g/L K_2_HPO_4_ per liter, 100 μg/mL PVK. The PVK solution was added to the MSM as the sole carbon source. The LB agar medium used for isolating PVK-degrading bacteria was prepared by adding 1.5% (*w*/*v*) agar powder in the LB medium. All media were autoclaved at 121 °C for 15 min.

### 2.3. Enrichment and Acclimatization of Bacterial Strains

Enrichment of the bacterial strains was carried out through a series of dilution and incubation. First, one gram (1 g) of collected soil was suspended in 9 mL of sterile distilled water and agitated for one minute. This homogenized soil extract solution was then diluted by adding 1 mL of the solution to 9 mL of sterile purified water, and the process was repeated six times to achieve a 10^6^-fold dilution. One milliliter of the diluted solution was then added to 49 mL of sterile LB medium containing 20 μg/mL PVK antibiotic as the sole carbon source, incubated at 30 °C, shaken at 150 rpm for 6 or 7 days until an optimal optical density (OD) is reached.

The cultures were then exposed to gradually increased PVK concentrations (40 to 100 μg/ml) to acclimatize the bacterial strains to PVK antibiotic. Specifically, 2 mL of enriched culture medium was transferred to the first flask containing 48 mL of LB medium with 40 μg/mL of PVK; then 3 mL of the solution was transferred from the first flask to a second flask containing 47 mL of LB medium with 60 μg/mL of PVK; next, 4 mL of solution was transferred from the second flask to a third flask containing 46 mL of LB medium with 80 μg/mL of PVK; and finally 5 mL of solution was transferred from the third flask to a fourth flask containing 45 mL of LB medium with 100 μg/mL of PVK. The above transfers were performed at 4-day intervals. In other words, the bacterial strains were given 4 days to acclimatize to each PVK concentration from 40 to 100 μg/mL [18]. The purpose of acclimatization was to obtain bacterial strains with high tolerance and degradation ability to PVK antibiotics.

### 2.4. Isolation and Purification of Enriched Bacterial Strains

Isolation and purification were performed after enrichment and acclimatization. Specifically, the solution from the final flask of acclimatization was serially diluted with sterile distilled water for three times (i.e., 1:1000 dilution), spread onto the LB agar medium, and incubated at 30 °C for 48 h. A streak plate method was used to carry out the separation and purification of the bacteria, with the specific operation steps outlined as follows: (i) An inoculating loop was used to transfer the bacterial culture onto the LB agar medium to perform a four-line streak on the plate. Specifically, moving the inoculating loop backward on the medium surface to allow the culture solution on the inoculation loop to gradually dilute and disperse along the pre-drawn lines on the medium surface; and (ii) a single cell was then cultured and each cell grew into a colony. The above steps were repeated three times to obtain pure cultures.

### 2.5. Qualitative Screening of PVK-Degrading Bacteria

To qualitatively determine if an isolated bacterial strain was capable of degrading PVK, a piece of filter paper containing 10 μg of PVK was applied to the surface of the agar which had been inoculated with a bacterial strain. As the diffusion distance of PVK in the agar increased, the PVK concentration decreased logarithmically to a certain concentration below which the bacterium would not grow, thus forming a transparent antimicrobial circle on the filter paper. The size of this inhibition zone reflected the sensitivity of the test bacteria to PVK, i.e., the smaller the circle, the more effective the bacterium is in degrading PVK.

### 2.6. Qquantitative Evalatution of PVK-Degrading Bacteria

The isolated bacterial strains that had shown the ability of degrading PVK in the above-discussed qualitative screening were further evaluated quantitatively for their effectiveness by using the thiol mercuric salt UV spectrophotometry (TMSUS) [17]. Specifically, the purified strains were inoculated on an LB medium containing 100 μg/mL of PVK in a shaker culture at a dose of 1%, and the concentration of survived PVK in the medium was measured by TMSUS in 48 h.

**Figure 1:**
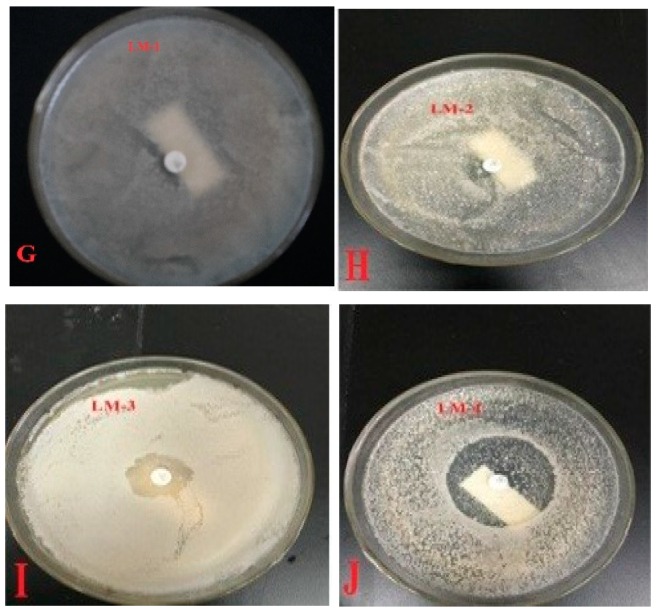
Results of penicillin V potassium (PVK) susceptibility test. G: LM-1; H: LM-2; I: LM-3; J: LM-4.

The two key steps in TMSUS measurement were: (i) To find the maximum detection wavelength for PVK, and (2) to establish a standard curve to correlate the PVK concentration to the measured absorbance value. To determine the maximum detection wavelength, 25 mL of PVK solution of 100 μg/mL was prepared in a 50 mL volumetric bottle. Following the procedure described in [18], the absorbance was measured in the range from 310 to 340 nm. When a peak appeared, the scan gradient was narrowed in the wavelength range around the peak to find the exact maximum absorption wavelength. The measured result is shown in Figure 1 and it was found that the maximum detection wavelength was around 325 nm for PVK, which is consistent with the range of 324 to 345 nm reported in the literature for the maximum absorption of penicillin antibiotics [17,19].

To establish the standard curve, PVK solutions with concentrations of 0, 15, 30, 45, 60, 75, and 90 μg/mL were prepared and the absorbance of PVK was measured at the maximum absorption wavelength (325 nm). Distilled water was used as the blank control (0 concentration). There was a good linear relationship between the PVK concentration and the absorbance value. The regression equation of the standard curve was determined to be Y = 0.006X − 0.001, with R^2^ = 0.998, where Y is the absorbance and X is the PVK concentration in μg/mL.

### 2.7. Morphological Observation of Isolated Strains

Gram staining was used to observe the morphology of the isolated bacterial strains as a means of preliminary identification, following the method reported in [20]. The observed morphological features included color, opaqueness, and surface texture of the bacterial colony. These features would help visual identification of bacterial strains.

### 2.8. Gene Sequencing and Phylogenetic Analysis of Isolated Strains

The PVK-degrading isolates were cultivated on the LB agar medium at 30 °C for 48 h. The culture was used for the amplification of bacterial 16S rRNA gene by PCR. Two universal 16S rRNA gene primers (F27:5′-AGTTTGATCMTGGCTCAG-3′ and R1492: 5′-GGTTACCTTGTTACGACTT-3′) were employed. Culture samples of 25 μL were prepared and each sample was composed of 0.5 μL of bacterial culture as the template DNA, 7.5 μL of 2× Taq PCR Master Mix (containing 0.2 U Taq DNA polymerase/μL, 250 μM of deoxy-ribonucleoside triphosphate (dNTP), 2.5 μL of 10× Buffer with Mg^2+^ (Takara, Tianjin, China), 0.5 μL of primer (10 μM), and 19.8 μL of double-distilled H_2_O. The PCR procedure was carried out as follows: Primary denaturation at 94 °C for 4 min; 30 cycles of denaturation at 94 °C for 45 s; annealing at 55 °C for 45 s; and extension at 72 °C for 60 s; and an additional reaction for 10 min at 72 °C. The PCR products were detected on 1.5% agarose gel to confirm its purity and size. The PCR products were sent to Tiangen Biotech (Beijing, China) for sequencing.

The 16S rRNA gene sequences were compared with other 16S rRNA gene sequences available in Genbank by using the Basic Local Alignment Search Tool (BLASTN) program and aligned with similar sequences by using multiple sequence alignment software [21].The phylogenetic tree was constructed by applying the neighbor-joining method using Molecular Evolutionary Genetics Analysis (MEGA) 7.0 program based on Kimura-2 parameters with 1000 replicates of bootstrap values [22].

## 3. Results and Discussion

### 3.1. Isolation and Initial Screening of PVK-Degrading Bacteria

A total of four PVK-degrading bacterial strains (LM-1, LM-2, LM-3, LM-4) were isolated from the soil collected on the pig farm. All the isolates were first subjected to preliminary screening which was carried out through antibiotic susceptibility tests against PVK. The inhibitory zone diameters of LM-1, LM-2, LM-3, and LM-4 were 12.42 ± 1.58 mm (mean ± standard deviation, SD), 18.37 ± 3.01 mm, 16.32 ± 1.06 mm, and 35.61 ± 1.53 mm, respectively (Figure 1). The inhibitory zone diameter of LM-4 was significantly larger than that of the other three strains (LM-1, LM-2, and LM-3) (*p* < 0.05), and there were no statistically significant differences among LM-1, LM-2, and LM-3. Therefore, LM-4 was considered to be not effective in degrading PVK, and the other three bacterial strains were subjected to quantitative evaluation of TMSUS.

### 3.2. TMSUS Measurements

The measured absorbance values for the three bacterial strains (LM-1, LM-2, LM-3) are summarized in Table 1, along with the blank controls. Using the standard curve (Y = 0.006X − 0.001), these absorbance values were converted to PVK concentrations (survived PVK after exposing to the three degrading strains). Comparing with the corresponding control, it was apparent that all three isolated strains were capable of degrading PVK, with degradation rates of 68%, 66%, and 38% for strains LM-1, LM-2, and LM-3, respectively (Table 1). Statistical analysis indicated that the degradation rates of LM-1 and LM-2 were significantly higher than that of LM-3 (*p* < 0.05), while there was no statistically significant difference between LM-1 and LM-2 (*p* > 0.05). Therefore, LM-3 was de-selected and further analysis was performed to characterize strains LM-1 and LM-2.

### 3.3. Morphological Features of Isolated Strains

**Figure 2:**
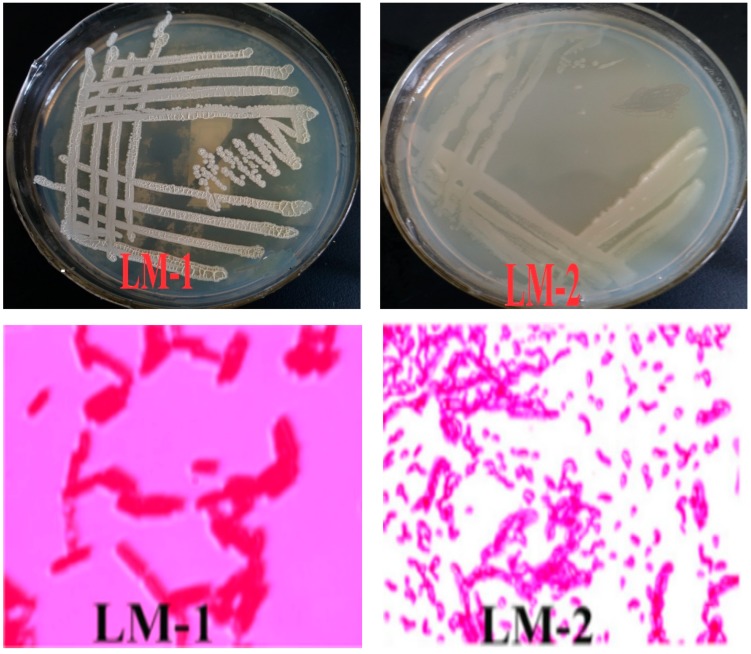
Individual forms of bacterial isolates of LM-1 and LM-2.

The colonial morphology of LM-1 strain could be described as follows: White, opaque, circular, wrinkled in surface, and convex in center. The colonial morphology of LM-2 strain was observed as milky white, opaque, circular, and smooth on the surface, and neat in the edge (Figure 2). The morphological features of the two colonies suggested that both LM-1 and LM-2 were Gram-positive bacteria [20]; LM-1 was linearly arranged rods and the LM-2 was dull circles (short but straight) (Figure 2).

### 3.4. Gene Sequencing and Phylogenetic Analysis of Two PVK-Degrading Isolates

**Figure 3:**
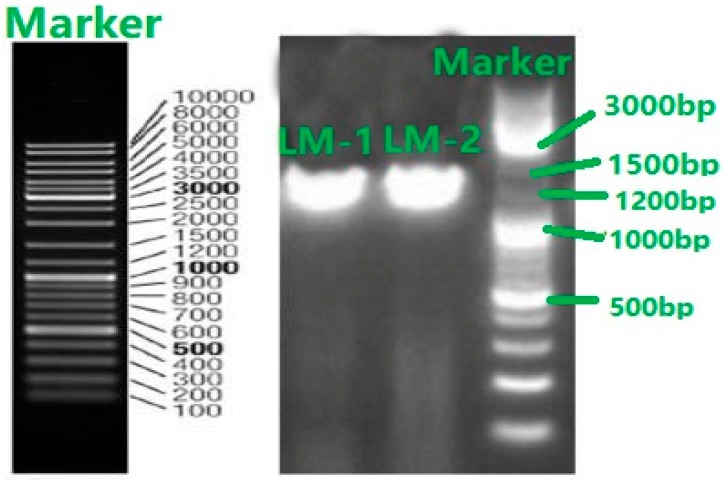
Results of PCR amplification of two bacterial isolates LM-1 and LM-2.

**Figure 4:**
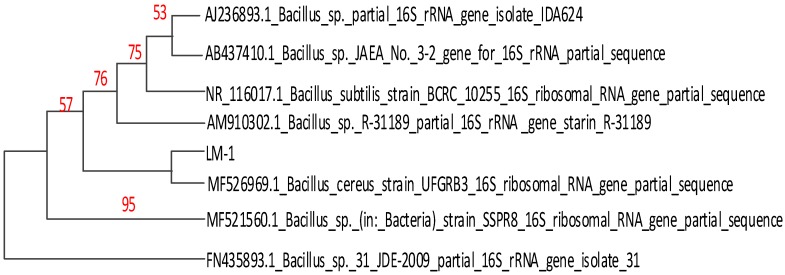
Phylogenetic analysis of isolated strain LM-1 in the neighbor-joining tree.

**Figure 5:**
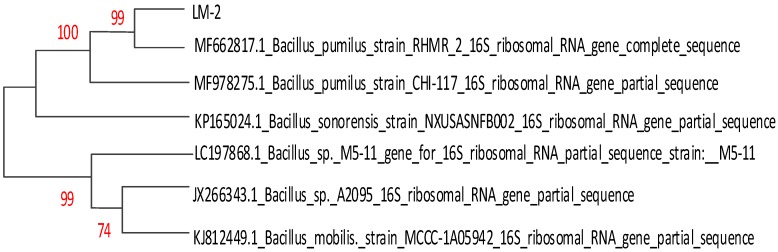
Phylogenetic analysis of isolated strain LM-2 in the neighbor-joining tree.

The 16S rDNA of LM-1 and LM-2 isolates were amplified using the primers 27F and 1492R, resulting in a characteristic single band of approximately 1490 bp (Figure 3). The isolated strain LM-1 was identified as *Bacillus cereus* (*B. cereus*) subgroup on the basis of 16S rDNA gene sequencing (Figure 1S in Supplementary Materials) and the phylogenetic tree (Figure 4). The isolated strain LM-2 was identified as *Bacillus pumilus* (*B. pumilus*) subgroup on the basis of 16S rDNA gene sequencing (Figure 2S in Supplementary Materials) and the phylogenetic tree (Figure 5). The phylogenetic trees of the two isolates clearly demonstrated its evolutionary relationship with a group of *B.* species produced by a neighbor-joining method with the help of MEGA 7.0 program. The closest relative of LM-1 was *B. cereus* strain URGRB3 (100%), and the closest relative of LM-2 was *B. pumilus* strain RHMR 2 (100%).

Both LM-1 and LM-2 belonged to the genus *Bacillus*, more than 50 species of which have been identified. The genus *Bacillus* is Gram-positive, and much research has demonstrated the wide distribution of the genus *Bacillus* in activated sludge, plants, soils, and wastewater [23], which can produce spores that are resistant to adverse conditions. Research has shown that *Bacillus* can produce antimicrobial substances that inhibit the reproduction of harmful microorganisms and degrade the nutrients in the soil. For example, Peng [24] isolated a strain of *Bacillus pumilis* 4D-14 from broiler farms, which had strong inhibitory effect on multiple strains of pathogenic bacteria. Yang et al. [25] isolated a strain *Bacillus cereus* XG1 from the watermelon leaves infected by watermelon bacterial fruit rot and they concluded that the strain XG1 had the potential to be developed as a microbe-herbicide. Wang et al. [26] isolated a *Bacillus cereus* strain WHY-2 from the deep-sea surface sediments with an effective function of adsorbing thorium ions (Th), which could alleviate the environmental pollution caused by thorium ions as radioactive substances in groundwater.

The antibiotic resistant bacteria play a major role in the microbial degradation of antibiotics. These bacteria can produce corresponding degrading enzymes, which destroy the molecular structure of antibiotics by modification or hydrolysis [27]. Several studies (e.g., [28]) have found that antibiotic degrading enzymes mainly include the following four types: *β*-Lactamase, aminoglycoside modifying enzyme, macrolide passivase, and chloramphenicol inactivating enzyme, among which *β*-Lactamase can destroy the chemical bonds of cephalosporins and penicillins. Furthermore, there are ring-opening oxidases associated with fosfomycin resistance as well as esterases associated with macrolides resistance that can breach the structure of the corresponding antibiotics. Although the degradation mechanisms of PVK was not investigated in this study, it was speculated that the degradation of PVK by LM-1 and LM-2 isolated in this study was due to the production of *β*-Lactamase by the two isolates, which could damage the *β*-lactam ring structure of PVK and impel the proceeding of biodegradation.

## 4. Conclusions

Two PVK-degrading bacterial strains (LM-1 and LM-2) were isolated from the soil collected on a pig farm. The two bacterial strains were characterized as belonging to the genus *Bacillus*. The 16S rDNA sequence comparison indicated that LM-1 and *B. cereus* strain URGRB3 were members of the same genomic species, so were LM-2 and *B. pumilus* strain RHMR2. These two bacterial strains extended our knowledge of the list of *Bacillus* species that could utilize PVK antibiotic as the sole carbon source. LM-1 could degrade 68% of PVK-antibiotic in 48 h at 30 °C when the initial concentration of PVK was 100 ppm, and LM-2 could degrade 66% PVK-antibiotic under the same conditions. The strains *B. cereus* and *B. pumilus* may have potential for bioremediation of PVK-contaminated environment, such as soil and wastewater, but further research is needed to confirm the performance of these strains in field conditions.

## Figures and Tables

**Table 1:** Measured absorbance values and calculated penicillin V potassium (PVK) concentrations for three isolated strains.

Strains	PVK Concentration of Control Group (μg/mL, Mean ± SD)	OD_control_ (Mean ± SD)	PVK Concentration of Test Group (μg/mL, Mean ± SD)	OD_test_ (Mean ± SD)	RD (Mean ± SD)
LM-1	96.28 ± 2.55	0.57 ± 0.015	33.50 ± 5.78	0.18 ± 0.055	0.68 ± 0.091
LM-2	85.17 ± 1.67	0.50 ± 0.021	29.06 ± 12.62	0.17 ± 0.076	0.66 ± 0.14
LM-3	84.61 ± 12.50	0.51 ± 0.031	52.94 ± 4.84	0.32 ± 0.031	0.38 ± 0.095

## Supplementary Materials

The following are available online at https://www.mdpi.com/1660-4601/16/12/2166/s1, Figure 1S: 16S rDNA sequencing of bacterial isolate LM-1, Figure 2S: 16S rDNA sequencing of bacterial isolate LM-2.
