# Supplementary material for: Isolation, Screening, and Characterization of Antibiotic-Degrading Bacteria for Penicillin V Potassium (PVK) from Soil on a Pig Farm
Source: Int J Environ Res Public Health. 2019 Jun 19;16(12):2166. doi: 10.3390/ijerph16122166 (PMC6616527; doi:10.3390/ijerph16122166)
Supplement: Supplementary file 1 [file ijerph-16-02166-s001.pdf]

TGGCTCAGGATGAACGCTGGCGGCGTGCCTAATACATGCAAGTCGAGCGAATGGATTAAGAGCTTGCTCTTATGAAGTTAGCG
GCGGACGGGTGAGTAACACGTGGGTAACCTGCCCATAAGACTGGGATAACTCCGGGAAACCGGGGCTAATACCGGATAACATT
TTGAACTGCATGGTTCGAAATTGAAAGGCGGCTTCGGCTGTCACTTATGGATGGACCCGCGTCGCATTAGCTAGTTGGTGAGGT
AACGGCTCACCAAGGCAACGATGCGTAGCCGACCTGAGAGGGTGATCGGCCACACTGGGACTGAGACACGGCCCAGACTCCT
ACGGGAGGCAGCAGTAGGGAATCTTCCGCAATGGACGAAAGTCTGACGGAGCAACGCCGCGTGAGTGATGAAGGCTTTCGG
GTCGTAAAACTCTGTTGTTAGGGAAGAACAAGTGCTAGTTGAATAAGCTGGCACCTTGACGGTACCTAACCAGAAAGCCACGG
CTAACTACGTGCCAGCAGCCGCGGTAATACGTAGGTGGCAAGCGTTATCCGGAATTATTGGGCGTAAAGCGCGCGCAGGTGGT
TTCTTAAGTCTGATGTGAAAGCCCACGGCTCAACCGTGGAGGGTCATTGGAAACTGGGAGACTTGAGTGCAGAAGAGGAAAG
TGGAATTCCATGTGTAGCGGTGAAATGCGTAGAGATATGGAGGAACACCAGTGGCGAAGGCGACTTTCTGGTCTGTAACTGAC
ACTGAGGCGCGAAAGCGTGGGGAGCAAACAGGATTAGATACCCTGGTAGTCCACGCCGTAAACGATGAGTGCTAAGTGTTAG
AGGGTTTCCGCCCTTTAGTGCTGAAGTTAACGCATTAAGCACTCCGCCTGGGGAGTACGGCCGCAAGGCTGAAACTCAAAGG
AATTGACGGGGGCCCGCACAAGCGGTGGAGCATGTGGTTTAATTCGAAGCAACGCGAAGAACCTTACCAGGTCTTGACATCCT
CTGAAAACCCTAGAGATAGGGCTTCTCCTTCGGGAGCAGAGTGACAGGTGGTGCATGGTTGTCGTCAGCTCGTGTCGTGAGAT
GTTGGGTTAAGTCCCGCAACGAGCGCAACCCTTGATCTTAGTTGCCATCATTAAGTTGGGCACTCTAAGGTGACTGCCGGTGAC
AAACCGGAGGAAGGTGGGGATGACGTCAAATCATCATGCCCCTTATGACCTGGGCTACACACGTGCTACAATGGACGGTACAA
AGAGCTGCAAGACCGCGAGGTGGAGCTAATCTCATAAAACCGTTCTCAGTTCGGATTGTAGGCTGCAACTCGCCTACATGAAG
CTGGAATCGCTAGTAATCGCGGATCAGCATGCCGCGGTGAATACGTTCCCGGGCCTTGTACACACCGCCCGTCACACCACGAGA
GTTTGTAACACCCGAAGTCGGTGGGGTAACCTTTTTGGAGCCAGCCGCCTAAGGTGGGACAGATGATTGGGGTGAAGTCGTA
ACAA

Figure 1S. 16S rDNA sequencing of bacterial isolate LM-1.

TGGCTCAGGACGAACGCTGGCGGCGTGCCTAATACATGCAAGTCGAGCGGACAGAAGGGAGCTTGCTCCCGGA
TGTTAGCGGCGGACGGGTGAGTAACACGTGGGTAACCTGCCTGTAAGACTGGGATAACTCCGGGAAACCGGAG
CTAATACCGGATAGTTCCTTGAACCGCATGGTTCAAGGATGAAAGACGGTTTCGGCTGTCACTTACAGATGGAC
CCGCGGCGCATTAGCTAGTTGGTGAGGTAACGGCTCACCAAGGCGACGATGCGTAGCCGACCTGAGAGGGTGA
TCGGCCACACTGGGACTGAGACACGGCCCAGACTCCTACGGGAGGCAGCAGTAGGGAATCTTCCGCAATGGA
CGAAAGTCTGACGGAGCAACGCCGCGTGAGTGATGAAGGTTTTCGGATCGTAAAGCTCTGTTGTTAGGGAAGA
ACAAGTGCAAGAGTAACTGCTTGCACCTTGACGGTACCTAACCAGAAAGCCACGGCTAACTACGTGCCAGCAG
CCGCGGTAATACGTAGGTGGCAAGCGTTGTCCGGAATTATTGGGCGTAAAGGGCTCGCAGGCGGTTTCTTAAGT
CTGATGTGAAAGCCCCCGGCTCAACCGGGGAGGGTCATTGGAAACTGGGAAACTTGAGTGCAGAAGAGGAGA
GTGGAATTCCACGTGTAGCGGTGAAATGCGTAGAGATGTGGAGGAACACCAGTGGCGAAGGCGACTCTCTGGT
CTGTAACTGACGCTGAGGAGCGAAAGCGTGGGGAGCGAACAGGATTAGATACCCTGGTAGTCCACGCCGTAAA
CGATGAGTGCTAAGTGTTAGGGGGTTTCCGCCCCTTAGTGCTGCAGCTAACGCATTAAGCACTCCGCCTGGGGA
GTACGGTCGCAAGACTGAAACTCAAAGGAATTGACGGGGGCCCGCACAAGCGGTGGAGCATGTGGTTTAATTC
GAAGCAACGCGAAGAACCTTACCAGGTCTTGACATCCTCTGACAACCCTAGAGATAGGGCTTTCCCTTCGGGG
ACAGAGTGACAGGTGGTGCATGGTTGTCGTCAGCTCGTGTCGTGAGATGTTGGGTTAAGTCCCGCAACGAGCG
CAACCCTTGATCTTAGTTGCCAGCATTCAGTTGGGCACTCTAAGGTGACTGCCGGTGACAAACCGGAGGAAGG
TGGGGATGACGTCAAATCATCATGCCCCTTATGACCTGGGCTACACACGTGCTACAATGGACAGAACAAAGGGC
TGCGAGACCGCAAGGTTTAGCCAATCCCACAAATCTGTTCTCAGTTCGGATCGCAGTCTGCAACTCGACTGCGT
GAAGCTGGAATCGCTAGTAATCGCGGATCAGCATGCCGCGGTGAATACGTTCCCGGGCCTTGTACACACCGCCC
GTCACACCACGAGAGTTTGCAACACCCGAAGTCGGTGAGGTAACCTTTATGGAGCCAGCCGCCGAAGGTGGG
GCAGATGATTGGGGTGAAGTCGTAACAA

Figure 2S. 16S rDNA sequencing of bacterial isolate LM-2.
